# The assessment of executive functions to test the integrity of the nigrostriatal network: A pilot study

**DOI:** 10.3389/fpsyg.2023.1121251

**Published:** 2023-03-29

**Authors:** Ciro Rosario Ilardi, Girolamo di Maio, Ines Villano, Giovanni Messina, Vincenzo Monda, Antonietta Messina, Chiara Porro, Maria Antonietta Panaro, Nadia Gamboz, Alessandro Iavarone, Marco La Marra

**Affiliations:** ^1^IRCCS Synlab SDN, Naples, Italy; ^2^Department of Experimental Medicine, University of Campania “Luigi Vanvitelli”, Naples, Italy; ^3^Department of Clinical and Experimental Medicine, University of Foggia, Foggia, Italy; ^4^Department of Movement Sciences and Wellbeing, University of Naples “Parthenope”, Naples, Italy; ^5^Department of Biosciences, Biotechnologies and Biopharmaceutics, University of Bari, Bari, Italy; ^6^Laboratory of Experimental Psychology, Suor Orsola Benincasa University, Naples, Italy; ^7^Neurological Unit, CTO Hospital, AORN “Ospedali dei Colli”, Naples, Italy

**Keywords:** Parkinson’s disease, executive functions, frontal assessment battery-15, diagnosis, motor symptoms

## Abstract

**Background:**

Parkinson’s disease (PD) is a chronic neurodegenerative disorder characterized by motor and non-motor symptoms. The latter mainly include affective, sleep, and cognitive deficits. Non-demented PD patients often demonstrate impairments in several executive domains following neuropsychological evaluation. The current pilot study aims at assessing the discriminatory power of the Frontal Assessment Battery-15 (FAB15) in differentiating (i) non-demented PD patients and healthy controls and (ii) PD patients with more and less pronounced motor symptoms.

**Methods:**

Thirty-nine non-demented early-stage PD patients in the “on” dopamine state (26 females, mean age = 64.51 years, SD = 6.47, mean disease duration = 5.49 years, SD = 2.28) and 39 healthy participants (24 females, mean age = 62.60 years, SD = 5.51) were included in the study. All participants completed the FAB15. Motor symptoms of PD patients were quantified *via* the Unified Parkinson’s Disease Rating Scale-Part III (UPDRS-Part III) and Hoehn and Yahr staging scale (H&Y).

**Results:**

The FAB15 score, adjusted according to normative data for sex, age, and education, proved to be sufficiently able to discriminate PD patients from healthy controls (AUC = 0.69 [95% CI 0.60–0.75], SE = 0.06, *p* = 0.04, optimal cutoff = 11.29). Conversely, the battery lacked sufficient discriminative capability to differentiate PD patients based on the severity of motor symptoms.

**Conclusion:**

The FAB15 may be a valid tool for distinguishing PD patients from healthy controls. However, it might be less sensitive in identifying clinical phenotypes characterized by visuospatial impairments resulting from posteroparietal and/or temporal dysfunctions. In line with previous evidence, the battery demonstrated to be not expendable in the clinical practice for monitoring the severity of PD-related motor symptoms.

## 1. Introduction

Parkinson’s disease (PD) is a progressive neurodegenerative disorder whose etiology, resulting from the interaction between environmental and genetic factors, is still unclear (Hirsch et al., 2013; Beitz, 2014; Tysnes and Storstein, 2017; Simon et al., 2020). PD prevalence is about 1–4% (de Lau and Breteler, 2006; Pringsheim et al., 2014; Marras et al., 2018) while incidence is hovering around 16–19/100,000 new cases per year (Tanner and Goldman, 1996; Twelves et al., 2003; Alves et al., 2008).

The pathognomonic clinical manifestations of PD are bradykinesia and/or hypokinesia, rigidity, rest tremor, gait dysfunctions, and postural instability (Jankovic, 2005, 2008; National Collaborating Centre for Chronic Conditions (UK), 2006; Postuma et al., 2015). The pathophysiology of PD is mainly characterized by degeneration of dopaminergic neurons in the substantia nigra pars compacta (SNc) (Rodriguez-Oroz et al., 2009; Dirnberger and Jahanshahi, 2013; Pagonabarraga et al., 2015). This leads to functional alterations within the nigrostriatal pathway connecting the SNc to the dorsal striatum (Deumens et al., 2002; Di Monte, 2003; Herrera et al., 2017). A reduced supply of dopamine to the caudate nucleus affects visuomotor control (Yanagisawa et al., 1989; Owen, 1998; Bloem et al., 2004; Brooks and Piccini, 2006; Chieffi et al., 2014, 2019), making the patient unable to respond to external stimuli with rapid and appropriate intentional motor responses. In addition, decreased dopamine supply to the putamen results in the inability to perform fine sequential movements (Graybiel et al., 1990; Yu et al., 2013; Ilardi et al., 2022a; La Marra et al., 2022d).

Although the clinical management of PD is typically centered on the treatment of the cardinal motor symptoms, these often coexist with non-motor symptoms such as fatigue (Friedman et al., 2007; Herlofson and Kluger, 2017; Siciliano et al., 2018), depression (Cummings, 1992; Schrag, 2006; Reijnders et al., 2008), anxiety (Richard et al., 1996; Richard, 2005; Ray and Agarwal, 2020), apathy (den Brok et al., 2015; Pagonabarraga and Kulisevsky, 2017; D’Iorio et al., 2018), sleep disorders (Comella, 2007; Zhang et al., 2020; Maggi et al., 2021), urological dysfunctions (Blackett et al., 2009; Yeo et al., 2012; Margolesky et al., 2020), and cognitive impairments (Padovani et al., 2006; Litvan et al., 2012; Yang et al., 2016) ranging from mild deficits to overt dementia.

In PD, deficits of executive functions are the most representative expression of cognitive impairment in both demented and non-demented patients (Zgaljardic et al., 2003; Kudlicka et al., 2011; Dirnberger and Jahanshahi, 2013; Mack and Marsh, 2017; O’Callaghan and Lewis, 2017) and are likely due to abnormal activity in the frontostriatal network (Zgaljardic et al., 2003; Mack and Marsh, 2017; Lang et al., 2019). Executive functions are a family of top-down mental processes, such as attention, abstract thinking, planning, cognitive flexibility, inhibitory control and working memory, which are involved in the execution of complex action schemes aimed at adaptively coping with environmental requests in unfamiliar or conflicting contexts (Burgess and Simons, 2005; Diamond, 2013; La Marra et al., 2022b). Besides executive blunting often revealed during formal neuropsychological evaluation, it seems that PD patients are impaired in dual-task performance, i.e., the concurrent execution of two or more attention-demanding tasks, and compensate their difficulties in making simple procedural motor tasks, such as walking, *via* hypercontrolled movements (Kelly et al., 2012; Raffegeau et al., 2019). These phenomena can be considered indexes of a limited/overloaded executive control system (Ceravolo et al., 2012; Dirnberger and Jahanshahi, 2013). Since executive dysfunctions are associated with inability to perform efficiently daily activities and poor quality of life in non-demented PD patients (Ceravolo et al., 2012), early detection of executive deficits may enable clinicians to predict which patients will develop dementia.

Among the available psychometric tests devoted to the assessment of executive functions, the Frontal Assessment Battery (FAB) (Dubois et al., 2000) is the most widely used screening tool. It is employed internationally for assessing general executive functioning at the bedside and in the outpatient clinical practice. Some studies have found that non-demented PD patients got lower FAB scores than healthy controls (Lima et al., 2008; Kenangil et al., 2010; Koerts et al., 2011; Bezdicek et al., 2017). However, it is unclear whether the battery holds sufficient discriminatory power for distinguishing between non-demented PD patients and cognitively-intact individuals without PD (Hurtado-Pomares et al., 2018b). Furthermore, to the best of our knowledge, no study assessed the extent to which the FAB could differentiate PD patients according to the severity of motor symptoms. The close relationship between executive and motor functions is inherent in the very concept of executive functions and likely justified by shared neural mechanisms involving cortical and subcortical frontal regions (Raz, 1997; Rycroft et al., 2019) as well as basal ganglia (Dubois et al., 2000; Bezdicek et al., 2017) and cerebellum (Koppelmans et al., 2017). Interestingly, according to a recent theoretical model described by Koziol et al. (2012) and Koziol and Lutz (2013), the functional architecture of the brain would evolve from childhood to adulthood for promoting action control, facilitating interactive adaptive behaviors rather than the efficiency of cognitive processes. Hence, the maturation of frontal (and executive) functions may be significantly affected by the sensorimotor interaction with the environment.

The present pilot study aims to evaluate the above psychometric dimensions of the FAB’s clinicometric validity, but on a shortened version of the battery, namely, the Frontal Assessment Battery-15 (FAB15) (Ilardi et al., 2022b). The choice of this FAB version stems from its robust psychometric architecture that boasts a solid factorial structure, good internal consistency, excellent interrater and test-retest reliabilities, and regression-based norms extracted –to the best of our knowledge– from the largest normative sample ever recruited in Italy for a neuropsychological tool (Ilardi et al., 2022b). In addition, the FAB15 solves the well-known problem concerning the pronounced ceiling effect typically encountered in both healthy (Asaadi et al., 2016; Bezdicek et al., 2017; Hurtado-Pomares et al., 2018b; Goh et al., 2019; Abrahámová et al., 2022) and clinical populations (Stamelou et al., 2015; Hurtado-Pomares et al., 2018b; Goh et al., 2019) on the sixth and last FAB subtest, which was originally devised to explore one the components of the environmental dependency, i.e., the prehension behavior (Dubois et al., 2000). This makes the FAB15 more valid and severe as compared with the conventional six-item battery.

## 2. Materials and methods

### 2.1. Participants

Retrospective data collection was performed for consecutive patients referred to the Neurology Outpatient Clinic of CTO Hospital (Neurological Unit, AORN “Ospedali dei Colli,” Naples, Italy). Eligible patients satisfied the following inclusion criteria: diagnosis of PD according to MDS-PD Criteria (Postuma et al., 2015), ≤80 years of age, ≥5 years of formal schooling, no visual or hearing impairment, and adjusted scores higher than normative datasets provided for the Mini-Mental State Examination (MMSE). Patients were excluded if affected by atypical or secondary parkinsonism, Mild Cognitive Impairment (MCI) in PD (Litvan et al., 2012) or PD dementia (Goetz et al., 2009). In addition, we excluded patients with current or past history of major depression, bipolar disorder, schizophrenia, epilepsy, transient ischemic attack, stroke, head injury, serious medical illnesses, or alcohol/drug abuse. All PD patients were examined in the “on” dopamine state.

Healthy volunteers were recruited in order to construct a matched-control sample. Inclusion criteria were age ≤80 years, ≥5 years of education, no visual or hearing impairment, and adjusted MMSE score within the normality range. Exclusion criteria were previous or current neurocognitive (mild and major), psychiatric, or psychological disorders, and ongoing intake of psychotropic drugs potentially interfering with the efficiency of cognitive processes.

### 2.2. Materials and procedure

All participants were administered the MMSE and the FAB15. The latter is a validated shortened version of the conventional FAB, from which the prehension behavior subtest (environmental dependency) was removed following a thorough psychometric investigation on 1,187 healthy individuals (Ilardi et al., 2022b). Thus, the FAB15 consists of five subtests (i.e., similarities, phonological verbal fluency, Luria’s fist-edge-palm test, conflicting instructions, and go-no-go test) assessing as many executive domains (i.e., abstraction ability, cognitive flexibility, planning and executing motor sequences, sensitivity to interference, and inhibitory control). The total FAB15 score ranges from 0 to 15, with a higher score indicating a better executive functioning. The psychometric properties of the new FAB15 are described in detail elsewhere (Ilardi et al., 2022b).

For PD patients, we had available data about disease duration, Levodopa Equivalent Daily Dose (LEDD) and severity of motor symptoms, which was assessed *via* both the Unified Parkinson’s Disease Rating Scale-Part III (UPDRS-Part III) (Goetz et al., 2008) and Hoehn and Yahr staging (H&Y scale) (Goetz et al., 2004). The UPDRS-Part III is composed of 18 items (response set from 0 = “normal” to 4 = “severe”) exploring motor signs of PD (i.e., speech, facial expression, rigidity, finger taps, hand movements, pronation/supination, toe tapping, leg agility, arising from chair, gait, freezing of gait, postural stability, posture, global spontaneity of movements, postural tremor of hands, kinetic tremor of hands, rest tremor amplitude, constancy of rest tremor). The H&Y scale is a descriptive clinical staging scale for PD and takes into account both functional and motor impairments. According to the H&Y scale, patients can be classified as having from a stage 1 (“unilateral involvement only”) to a stage 5 disease (“wheelchair bound or bedridden unless aided”). For all participants, the neuropsychological examination was conducted by trained neuropsychologists.

### 2.3. Ethics statement

All participants gave prior written informed consent to the study which was approved by the ethics committee of the University of Campania “Luigi Vanvitelli” and carried out according to the 1964 Declaration of Helsinki.

### 2.4. Statistical analyses

For descriptive purposes, between-group comparisons were conducted by means of two-way chi-squared test (χ^2^) for nominal variables, and univariate analysis of variance (ANOVA), Mann–Whitney (U) Test, or Kruskal–Wallis (H) test for continuous variables, when appropriate. To determine the diagnostic accuracy of FAB15, we firstly adjusted raw scores according to normative correction grids for sex, age, and education (Ilardi et al., 2022b). Then, we ran three non-parametric Receiver Operating Characteristic (ROC) curve analyses (Mandrekar, 2010), where group (PD patients vs. healthy controls) and severity of motor symptoms (PD patients with greater motor symptoms vs. PD patients with minor motor symptoms) entered as state variables. For all ROC curve analyses, the FAB15 corrected score entered as test variable. The Youden index (YI, sensitivity + specificity − 1) was employed in order to identify any optimal cutoffs (Liu, 2012; Unal, 2017). A *p*-value ≤ 0.05 was considered statistically significant. Analyses were conducted by means of IBM SPSS Statistics for Windows, v. 26 (IBM, Armonk, 204 NY, USA) and easyROC, v. 1.3.1., using R language.

## 3. Results

### 3.1. *A priori* power analysis

At a nominal alpha level of 0.05, 1–β set to 0.80, expected AUC of 0.80, and allocation ratio = 1, the required total sample size for conducting a ROC curve analysis was 20 sample units (Obuchowski, 2005).

### 3.2. Descriptive statistics

We analyzed data from 39 PD patients (26 females, *M* age = 64.51 years, *SD* = 6.47, *M* education = 9.97 years, *SD* = 4.25; *M* disease duration = 5.49 years, *SD* = 2.28) and 39 healthy participants (24 females, *M* age = 62.60 years, *SD* = 5.51, *M* education = 8.97 years, *SD* = 3.61). General descriptive statistics are summarized in Table 1. No between-group differences were detected in the frequency of the sex variable’s levels [χ^2^_(1)_ = 0.233, *p* = 0.64]. Similarly, patient and control groups were equivalent in terms of years of age [*F*_(1,76)_ = 1.966, *p* = 0.165], years of education [*F*_(1,76)_ = 1.256, *p* = 0.266], and MMSE score [*F*_(1,76)_ = 0.02, *p* = 0.89]. Instead, as expected, healthy subjects outperformed PD patients on general executive functioning [FAB15, *F*_(1, 76)_ = 4.464, *p* = 0.03]. Twenty-two patients got an adjusted FAB15 score below the upper limits of the equivalent score 1 (score < 11.12), suggesting a performance on the edge of normality.

**TABLE 1:** Descriptive statistics concerning the patient and control group.

Sample characteristics	PD patients	Scoring range		Healthy controls	Scoring range		*p*-value
		**Min**	**Max**		**Min**	**Max**	
Sex (f/m)^a^	26/13			15/24			ns
Age (years)^b^	64.51 (6.47)	52	74	62.60 (5.51)	52	75	ns
Education (years)^b^	9.97 (4.25)	5	18	8.97 (3.61)	5	18	ns
MMSE (raw)^b^	28.81 (0.95)	27	30	28.85 (0.93)	27	30	ns
FAB15 (raw)^b^	11.72 (2.27)	5	15	12.64 (1.51)	10	15	*
LEDD (mg)	325.00 (265.88)	100	800				
Disease duration (years)	5.49 (2.28)	2	10				
UPDRS-Part III	12.12 (6.57)	3	26				
H&Y scale	1.88 (0.60)	1	3				
H&Y stage 1	13						
H&Y stage 2	22						
H&Y stage 3	4						

PD, Parkinson’s disease; MMSE, mini-mental state examination; FAB15, frontal assessment battery-15; LEDD, levodopa equivalent daily dose; UPDRS-Part III, Unified Parkinson’s Disease Rating Scale-Part III; H&Y, Hoehn and Yahr staging; ns, not significant. Values are expressed as frequency for nominal variables and mean (SD) for continuous variables.

^a^χ^2^.

^b^ANOVA.

*p < 0.05.

### 3.3. ROC curve analysis: Group as state variable

To assess whether the FAB15 was able to differentiate PD patients from healthy controls, a ROC curve analysis was performed, entering the group (patients vs. controls) as state variable and the adjusted FAB15 score as test variable. The FAB15 proved to be sufficiently discriminative (AUC = 0.69 [95% CI 0.60–0.75], SE = 0.06, *p* = 0.04). Based on a simultaneous assessment of sensitivity and specificity across all the possible cutoff points, the optimal FAB15 cutoff for differentiating PD patients from healthy participants on the adjusted scores distribution was 11.29 (sensitivity = 0.51, specificity = 0.89, YI = 0.28).

### 3.4. ROC curve analysis: Severity of motor symptoms as state variable

To examine the capability of the FAB15 in discriminating between PD patients with more- and less-deep impacting motor symptoms, two ROC curve analyses were performed. Based on tertiles (*T*_*i*_) calculated along the UPDRS-Part III score distribution (*T*_1_ = 8, *T*_2_ = 15), patients were divided into three subgroups (see Table 2): slight motor impairment (SMI, 14 patients getting a score ≤ 8), mild motor impairment (MMI, 11 patients getting a score ranging from 9 to 14), and large motor impairment (LMI, 14 patients getting a score ≥ 15). This 3-level ordinal variable was used as state variable while the adjusted FAB15 score entered as test variable. Results suggested that FAB15 lacked sufficient discriminative capability if required to differentiate PD patients based on the severity of motor symptoms evaluated *via* UPDRS: SMI group vs. MMI group, AUC = 0.50 [95% CI 0.13–0.87], SE = 0.19, *p* = 0.99; SMI group vs. LMI group, AUC = 0.64 [95% CI 0.31–0.97], SE = 0.17, *p* = 0.42; MMI group vs. LMI group, AUC = 0.60 [95% CI 0.24–0.95], SE = 0.18, *p* = 0.58. As concerns the H&Y score, patients were splitted into two subgroups in an attempt to balance the sample size, with all the limits that the overt positive skewness entails: 13 patients with H&Y stage 1 disease and 26 patients with H&Y stage ≥ 2 disease (see Table 3). Similarly, this nominal variable entered the ROC curve analysis as state variable, whereas the adjusted FAB15 score was employed as test variable. Again, results showed that FAB15 was unable to detect the variability of motor impairment in PD patients according to the H&Y classification: AUC = 0.67 [95% CI 0.40–0.99], SE = 0.15, *p* = 0.23.

**TABLE 2:** Descriptive statistics on UPDRS-based groups.

Sample characteristics	SMI group (*n* = 14)	Scoring range		MMI group (*n* = 11)	Scoring range		LMI group (*n* = 14)	Scoring range		*p*-value
		**Min**	**Max**		**Min**	**Max**		**Min**	**Max**	
Sex (f/m)^a^	10/4			6/5			10/4			ns
Age (years)^b^	60.33 (4.27)	55	67	62.40 (7.02)	52	74	64.00 (5.37)	55	70	ns
Education (years)^b^	9.00 (3.63)	5	13	8.60 (2.88)	5	13	8.17 (5.04)	5	18	ns
MMSE (raw)^b^	29.33 (0.52)	29	30	28.60 (0.89)	28	30	28.83 (0.75)	27	30	ns
FAB15 (raw)^b^	12.67 (2.42)	5	15	12.80 (2.39)	9	15	13.00 (1.09)	12	14	ns
LEDD (mg)^b^	300.00 (0.00)			287.50 (342.48)	100	800	400.00 (360.55)	100	800	ns
Disease duration (years)^b^	4.50 (2.07)	2	7	4.80 (1.48)	3	7	4.83 (1.83)	3	10	ns

SMI, slight motor impairment; MMI, mild motor impairment; LMI, large motor impairment; MMSE, mini-mental state examination; FAB15, frontal assessment battery-15; LEDD, levodopa equivalent daily dose; ns, not significant. Values are expressed as frequency for nominal variables and mean (SD) for continuous variables.

^a^χ^2^.

^b^H test.

**TABLE 3:** Descriptive statistics on H&Y-based groups.

Sample characteristics	H&Y stage 1 (*n* = 13)	Scoring range		H&Y stage ≥ 2 (*n* = 26)	Scoring range		*p*-value
		**Min**	**Max**		**Min**	**Max**	
Sex (f/m)^a^	11/4			15/9			ns
Age (years)^b^	60.50 (4.04)	55	64	62.77 (5.83)	52	74	ns
Education (years)^b^	8.25 (3.95)	5	13	8.69 (3.88)	5	18	ns
MMSE (raw)^b^	29.25 (0.96)	28	30	28.85 (0.69)	27	30	ns
FAB15 (raw)^b^	13.75 (1.26)	12	15	12.54 (2.02)	5	15	ns
LEDD (mg)^b^	200.00 (141.42)	100	300	356.25 (287.15)	100	800	ns
Disease duration (years)^b^	3.25 (0.96)	2	4	5.15 (1.68)	3	10	ns

H&Y, Hoehn and Yahr staging; MMSE, mini-mental state examination; FAB15, frontal assessment battery-15; LEDD, levodopa equivalent daily dose; ns, not significant. Values are expressed as frequency for nominal variables and mean (SD) for continuous variables.

^a^χ^2^.

^b^U test.

## 4. Discussion

A high proportion of PD patients without MCI/dementia demonstrate executive deficits following neuropsychological evaluation. These mainly involve planning and performing complex goal-directed actions, abstract thinking, inhibitory control, set-shifting, and working memory (Foster and Hershey, 2011; O’Callaghan et al., 2014; D’Iorio et al., 2021).

Dysfunction in the frontostriatal network is likely responsible for the onset of executive deficits in non-demented PD patients. The nigrostriatal pathology results in dopamine depletion in the dorsal striatum, with relative sparing of the ventral striatum. As a consequence, dopamine depletion in the somatosensory cortex –in addition to decreased connectivity within the somatosensory and dorsolateral prefrontal cortices (Tekin and Cummings, 2002; Helmich et al., 2010; Chieffi et al., 2017; Polito et al., 2020a; Villano et al., 2021)– accounts for the emergence of executive deficits in PD (Jellinger, 2001; O’Callaghan et al., 2014; Lang et al., 2019). Since executive functions orchestrate most of our daily activities (Bell-McGinty et al., 2002; Miller and Wallis, 2009; Stuss, 2011; Francavilla et al., 2020; Polito et al., 2020b; La Marra et al., 2022a,e), early identification of executive deficits in PD may help predict patients who will develop dementia.

In the current pilot clinicometric study, we tested the diagnostic properties of the short FAB15 in discriminating between non-demented PD patients and healthy controls. We found that non-demented PD patients showed normal global cognitive functioning but a certain degree of impairment in executive abilities. Furthermore, the FAB15 showed a fair diagnostic capability in differentiating between non-demented PD patients and healthy participants. As a consequence, we argue that FAB15 may be considered a valid tool to support diagnosis of PD, even independently on disease duration or severity of motor symptoms. This result is in line with previous evidence suggesting that (a) non-demented PD patients typically show executive blunting and (b) the FAB score is related to lower gray matter density in cortical regions strictly involved in executive functioning (Bezdicek et al., 2017; Ilardi et al., 2022a).

Nevertheless, in the context of PD, the FAB15 diagnostic capability is disputable but improvable. On the one hand, our small sample size and the patients’ clinical characteristics are to be accounted for (La Marra et al., 2022c); on the other hand, PD patients may also suffer from occipito-temporal dysfunctions determining visuospatial deficits (Watson and Leverenz, 2010; Koerts et al., 2011). From a neurocognitive standpoint and in line with the dual syndrome hypothesis, there would be two kinds of PD neuropsychological profiles: one with executive impairments reflecting neurofunctional abnormalities in the frontostriatal network, and the other one with visuospatial impairments due to posteroparietal (especially along the mesial portion, e.g., precuneus) and temporal dysfunctions (Kehagia et al., 2010, 2013). Since the FAB appears to be mainly sensitive to impairments in the dorsolateral, ventro-, and orbito-medial prefrontal regions, as well as in the basal ganglia (Dubois et al., 2000; Bezdicek et al., 2017; Hurtado-Pomares et al., 2018a), it might be able to identify non-demented PD patients with predominant executive but not visuospatial deficits (Bezdicek et al., 2017).

Our second aim was to check whether the battery was able to discriminate non-demented PD patients with more and less prominent motor marks, which were quantified by the UPDRS-Part III and H&Y scale. In sum, the FAB15 was unable to consistently capture the severity of motor symptoms caused by PD. Results from the present pilot study are supported by previous research finding no difference on the UPDRS score between groups of patients with higher and lower FAB scores (Matsui et al., 2006) and no correlation between motor and executive deficits in PD (Kenangil et al., 2010); in our study: FAB15 vs. UPDRS-Part III, *r*_rho_ = −0.104, *p* = 0.691; FAB15 vs. H&Y, *r*_rho_ = −0.105, *p* = 0.688.

By the very nature of pilot studies, there are critical limitations to their epistemological weight and interpretation. The main limitations of the present study have to be attributed to the narrow sample size and to the fact that patients were in the early stage of the disease. However, according to *a priori* power analysis, our sample appeared to be sufficiently large to test the FAB15 diagnostic accuracy. Moreover, the scientific literature of reference claims that cognitive deficits are typically detected in PD from the early stages after diagnosis (Poletti et al., 2012). However, it is certain that the discriminatory power of the FAB15 in relation to the severity of motor symptoms should be addressed with an *ad hoc* recruitment also covering an *a priori* classification of the disease severity levels.

It is important to note that our sample may not be sufficiently representative of the PD population since the occurrence of this pathology appears to be moderated by gender differences, with men affected twice as often as women (Baldereschi et al., 2000). Still, recent findings on PD have suggested that executive impairments are significantly greater in males than females after adjusting for demographic variables and disease severity (Reekes et al., 2020). This result was not replicated in our investigation (males with PD vs. females with PD on FAB15: *U* = 165.50, SE = 33.12, *p* = 0.92).

Finally, we included only PD patients in the “on” state. Nevertheless, it has been observed that, unlike PD-related motor symptoms, cognitive blunting may be not significantly affected by antiparkinsonian treatment with intravenous levodopa (Leh et al., 2010).

## 5. Conclusion

The FAB15 proved useful in differentiating PD patients without MCI/dementia and healthy controls. Conversely, this neuropsychological battery demonstrated to be not expendable in the clinical practice for monitoring the severity of PD-related motor symptoms. Our results should be taken with caution due to pilot study design. The clinicometric validity of the FAB15 needs to be further assessed in future investigations preferably encompassing a larger –and more differentiated– sample of PD patients.

## Data availability statement

The original contributions presented in this study are included in the article/supplementary material, further inquiries can be directed to the corresponding author.

## Ethics statement

All participants gave prior written informed consent to the study which was approved by the Ethics Committee of the University of Campania “Luigi Vanvitelli” and carried out according to the 1964 Declaration of Helsinki. The patients/participants provided their written informed consent to participate in this study.

## Author contributions

CRI and MLM: conceptualization, methodology, and formal analysis. CRI: software. AM and IV: validation. CRI, MLM, CP, AI, GdM, and VM: investigation. AM, GdM, IV, NG, and VM: resources. CRI, MP, and GdM: data curation. CRI, MLM, and IV: writing—original draft preparation. CRI, MLM, IV, and AI: writing—review and editing. NG: visualization. CRI, MLM, IV, GM, and AI: supervision and project administration. All authors read and agreed to the published version of the manuscript.
